# Effect of transcutaneous auricular vagus nerve stimulation on postoperative liver function in patients undergoing partial hepatectomy: a study protocol for a prospective, double-blind, randomized controlled trial

**DOI:** 10.3389/fmed.2025.1603543

**Published:** 2025-08-12

**Authors:** Yuanyuan Wang, Zhengxiu Sun, Yongao Lin, Mingshu Tao, Wenxin Zhao, Jinling Liu, Xiaoqin Guo, Chuyu Hang, Mingyuan Wang, Wen Tan, Xingyu Xiong, Jun-Li Cao, He Liu

**Affiliations:** ^1^Department of Anesthesiology, The Affiliated Hospital of Xuzhou Medical University, Xuzhou, China; ^2^NMPA Key Laboratory for Research and Evaluation of Narcotic and Psychotropic Drugs and Jiangsu Province Key Laboratory of Anesthesiology and Jiangsu Key Laboratory of Applied Technology of Anesthesia and Analgesia, Xuzhou Medical University, Xuzhou, China; ^3^Department of Anesthesiology and Clinical Research Center for Anesthesia and Perioperative Medicine and Key Laboratory of Anesthesia and Analgesia Application Technology, Huzhou Central Hospital, The Fifth School of Clinical Medicine of Zhejiang Chinese Medical University, Huzhou, China; ^4^Huzhou Central Hospital, The Affiliated Central Hospital Huzhou University, Huzhou, China; ^5^Affiliated Huzhou Hospital, Zhejiang University School of Medicine, Huzhou, China; ^6^Huzhou Key Laboratory of Basic Research and Clinical Translation for Neuromodulation, Huzhou Central Hospital, The Fifth School of Clinical Medicine of Zhejiang Chinese Medical University, Huzhou, China

**Keywords:** transcutaneous auricular vagus nerve stimulation, partial hepatectomy, perioperative hepatoprotection, neural modulation, organ protection

## Abstract

**Background:**

Partial hepatectomy remains a primary therapeutic intervention for various hepatic diseases. However, several intraoperative factors, including surgical manipulation, substantial blood loss, the need for blood transfusions, and hypoxic stress, can significantly impair liver function. Current perioperative strategies aimed at protecting the liver exhibit certain limitations. Transcutaneous auricular vagus nerve stimulation (taVNS), an emerging non-invasive neuromodulation technique, has demonstrated potential in preserving organ function through vagus nerve-mediated anti-inflammatory mechanisms. This study is designed to evaluate the hepatoprotective effects of taVNS on liver function in patients undergoing elective partial hepatectomy under general anesthesia.

**Methods/design:**

In this single-center, prospective, double-blind, randomized controlled trial, 140 patients scheduled for partial hepatectomy will be randomly allocated in a 1:1 ratio to either the transcutaneous auricular active-taVNS or sham taVNS groups. Both groups will receive 60-min stimulation sessions at four predefined time points: (1) at the onset of the first hepatic portal occlusion, (2) post-extubation, (3) on postoperative day 1 (6:00–7:00 a.m.), and (4) on postoperative day 2 (6:00–7:00 a.m.). The primary outcome is alanine aminotransferase (ALT) level measured in venous blood samples collected at 7:00 a.m. on postoperative day 2. Secondary outcomes include postoperative levels of inflammatory markers, renal function indicators, quality of recovery, gastrointestinal function recovery, pain, fatigue, anxiety, incidence of postoperative delirium, and time to first flatus, bowel movement, and oral intake, all of which will be assessed using validated instruments.

**Discussion:**

Postoperative liver function dysfunction following partial hepatectomy remains a significant clinical complication that negatively impacts patient prognosis and long-term survival outcomes. TaVNS, an emerging non-invasive neuromodulation technique, has demonstrated considerable potential for perioperative organ protection in preclinical studies. This study aims to provide robust evidence regarding the therapeutic efficacy of taVNS in reducing hepatic injury after partial hepatectomy. By introducing a novel approach to perioperative hepatic protection, taVNS may contribute valuable insights into the development of multimodal hepatoprotective strategies.

## 1 Introduction

Liver cancer ranks as the sixth most prevalent malignant tumor worldwide globally and is the third leading cause of cancer-related mortality (1). Epidemiological projections anticipate a 55% global increase in incidence, with 81% of new cases and 80% of related deaths concentrated in Asia and Africa. China, which bears one of the highest liver cancer burdens worldwide, faces substantial challenges in the prevention and management of the disease (2). Among liver tumors, benign hepatic hemangiomas are typically asymptomatic; however, when these tumors reach a significant size, they may cause compressive symptoms and potentially lead to severe complications, such as tumor rupture and life-threatening hemorrhage (3). Clinical studies with long-term follow-up of 236 patients diagnosed with hepatic hemangiomas demonstrated that 61% exhibited tumor growth, indicating a statistically significant progression rate (4). Progressively enlargement of hepatic hemangiomas is now considered an indication for surgical intervention (3). In patients presenting with localized hepatocellular carcinoma or hepatic hemangioma, partial hepatectomy allows for complete tumor resection while preserving adequate liver volume, thereby contributing to improved patient prognosis (5–7). However, several perioperative factors—including manipulation of the liver parenchyma during surgery, intermittent occlusion of the portal vein leading to hepatic ischemia–reperfusion injury (HIRI), intraoperative massive blood loss and transfusion, and chronic perioperative stress—can trigger and aggravate hepatic dysfunction, potentially leading to postoperative liver failure and increased mortality (5, 8, 9). A meta-analysis of 32 studies involving 19,503 patients reported considerable variability in post-hepatectomy complication rates. Specifically, minor hepatectomies were associated with hepatic dysfunction and severe complication rates ranging from 7.2 to 21.6%, whereas major hepatectomy showed an overall severe complication rate of 38.8%. In-hospital mortality was reported at 5.9%, with 30-day and 90-day mortality rates of 4.6 and 6.1%, respectively. Patients with compromised residual liver function and insufficient liver volume exhibit significantly rates of higher morbidity and mortality (10). A subsequent meta-analysis of 37 studies encompassing a total of 14,096 patients confirmed a significant association between postoperative severe hepatic dysfunction, liver failure, and decreased overall survival as well as disease-free survival (11). Therefore, the implementation of effective perioperative hepatoprotective strategies is of critical importance. In contemporary clinical practice, anesthesia preconditioning (APC) and ischemic preconditioning (IPC) are recognized as potential hepatoprotective interventions (12, 13). However, due to ongoing controversies regarding the clinical efficacy of IPC, its routine use remains limited. Notably, dexmedetomidine-based APC has shown potential in preserving hepatic function following partial hepatectomy, however, further validation through large-scale, multicenter clinical trial is necessary to confirm its long-term efficacy (12, 14, 15).

Preclinical studies suggest that vagus nerve stimulation (VNS) activates anti-inflammatory pathways, thereby exerting hepatoprotective effects (16–18). Research has shown that glutathione is a key antioxidant enzyme in the liver, the synthesis of which relies on the enzymatic activities of glutathione s-transferase (GST) and glutamate–cysteine ligase (GCL), formerly known as glutathione synthase (GSS). Evidence indicates that VNS enhances glutathione metabolism by modulating the GSS-GSH-GST axis, leading to a significant increase in the mRNA and protein expression of levels GSS and GST. As a result, VNS effectively elevates hepatic glutathione levels, enhances the liver’s intrinsic antioxidant capacity, and attenuates perioperative inflammatory responses (19). VNS activates the cholinergic anti-inflammatory pathway through the release of acetylcholine from vagus nerve efferent fiber terminals. Acetylcholine binds to *α*-7 nicotinic acetylcholine receptors (*α*-7nAChR) expressed on macrophages and Kupffer cells, thereby inhibiting their activation and reducing the production of pro-inflammatory cytokines such as interleukin-6 (IL-6) and tumor necrosis factor-*α* (TNF-α). This ultimately suppresses hepatic inflammation and alleviates liver injury. Notably, Kupffer cells also express α-7nAChR, exhibiting functional characteristics similar to those observed in macrophages (20, 21). Furthermore, studies suggest that stimulation of the hepatic branch of the vagus nerve following partial hepatectomy enhances hepatocyte proliferation by promoting the binding of acetylcholine to M3 receptors expressed on liver progenitor cells, thereby activating these cells and augmenting their proliferative and regenerative potential (22). Accumulating evidence suggests that the nuclear factor erythroid 2-related factor 2/heme oxygenase-1 (Nrf2/HO-1) signaling pathway confers protective effects on the liver. In a mouse model of hepatic ischemia–reperfusion, VNS at a frequency of 20 Hz induces the release of acetylcholine from vagal nerve terminals, which subsequently activates *α*-7nAChR and initiates the activation of the Nrf2/HO-1 pathway, leading to a significant reduction in hepatic inflammatory responses and ischemia–reperfusion injury (23).

Transcutaneous auricular vagus nerve stimulation (taVNS) is an innovative, cost-effective, and non-invasive neuromodulation technique. Research has demonstrated that taVNS achieves therapeutic outcomes comparable to those of traditional VNS across a range of clinical applications, including epilepsy, depression, chronic insomnia, and cerebral-cardiovascular protection. Given its favorable safety profile and ease of implementation, taVNS is increasingly recognized as a promising alternative therapeutic approach (24). In the field of clinical research, taVNS has demonstrated considerable promise in cerebrocardiovascular protection. Accumulating evidence indicates that taVNS administered during acute middle cerebral artery occlusion can reduce infarct volume and improve stroke outcomes through multiple mechanisms, including activation of the cholinergic anti-inflammatory pathway, suppression of excitotoxicity, and preservation of blood–brain barrier integrity (25, 26). Furthermore, a clinical study revealed that low-frequency taVNS (20 Hz) applied as a preconditioning intervention in patients with myocardial infarction, and continued for 2 h post-reperfusion, significantly reduced biomarkers of myocardial injury and levels of inflammatory cytokines (27, 28). The therapeutic potential of taVNS in protecting vital organs such as the heart and brain has been increasingly investigated. However, to date, no clinical studies have evaluated the impact of taVNS on hepatic injury.

Based on existing research, we propose the hypothesis that taVNS may offer hepatoprotective benefits during the perioperative period of partial hepatectomy. This study is designed to assess the efficacy of taVNS in preserving liver function in patients undergoing partial hepatectomy, with the objective of establishing a more robust theoretical basis for its clinical application and exploring a novel multimodal approach to perioperative liver protection.

## 2 Methods and analysis

### 2.1 Study design

**Figure 1:**
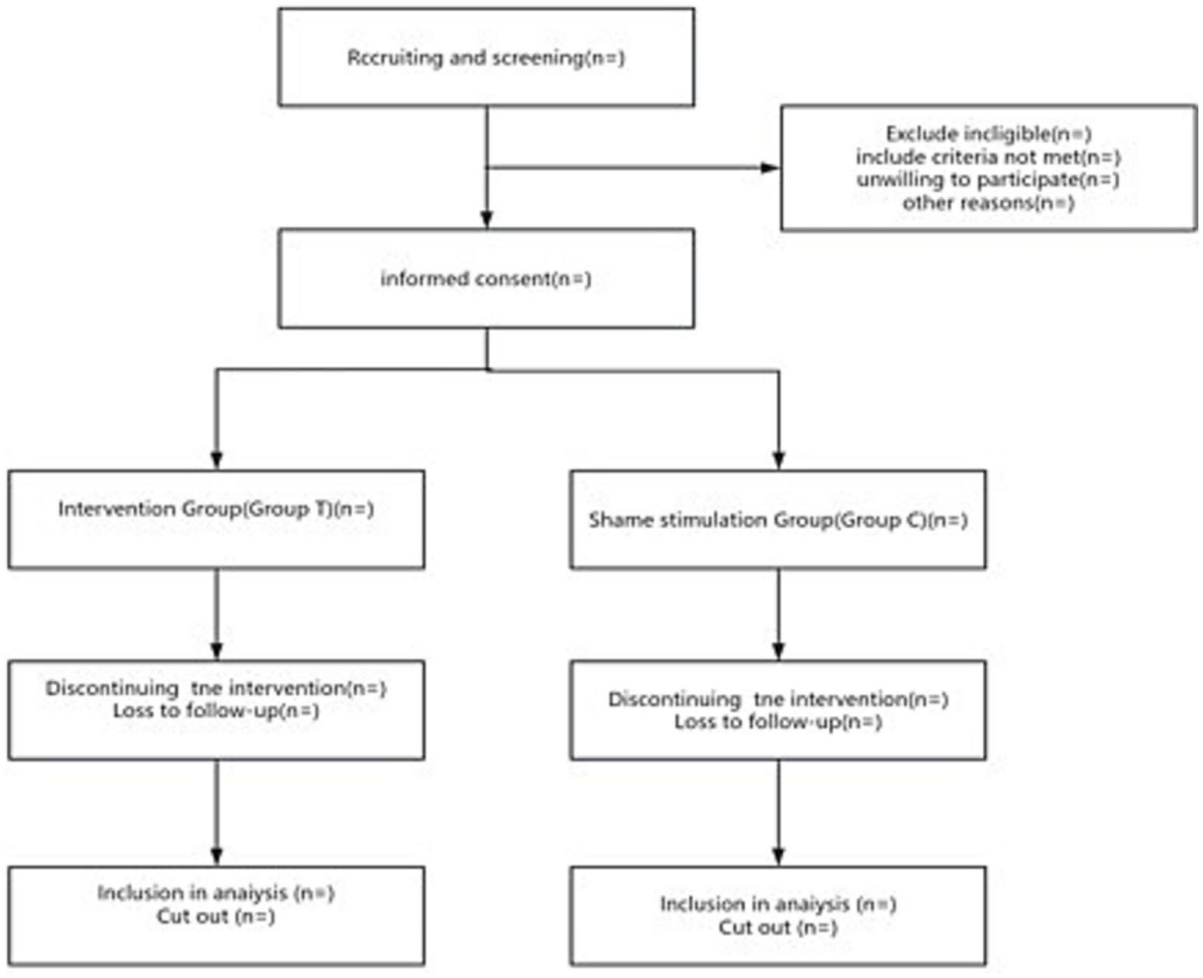
Flow diagram of the study.

This is a single-center, prospective, double-blind, randomized controlled clinical trial that will be conducted at the Affiliated Hospital of Xuzhou Medical University. A total of 140 patients scheduled for elective partial hepatectomy will be enrolled. The study protocol has received ethical approval from the Ethics Committee of the Affiliated Hospital of Xuzhou Medical University (approval number: XYFY2024-KL521-01) and has been registered with the Chinese Clinical Trial Registry (http://www.chictr.org.cn, Registration number: ChiCTR2400092005). Recruitment of participants is currently in progress, following strict adherence to predefined inclusion and exclusion criteria. Written informed consent will be obtained from all participants prior to enrollment. The study follows the Standard Protocol Items: Recommendations for Interventional Trials (SPIRIT) guidelines (Supplementary material 1) (29). The flow of patient recruitment, intervention, and outcome measurement is summarized in Figure 1.

### 2.2 Eligibility criteria

Patients eligible for partial hepatectomy will be recruited from the Affiliated Hospital of Xuzhou Medical University according to predefined inclusion and exclusion criteria, which will be assessed by the research team the day before surgery. All participants will provide written informed consent prior to undergoing baseline evaluation (Supplementary material 2).

### 2.3 Inclusion criteria

1. Age: 18–75 years
2. Scheduled for elective partial hepatectomy (liver cancer, hepatic hemangiomas)
3. American Society of Anesthesiologists (ASA) grade I-III
4. Able to comprehend study protocol and assessment scales
5. Capable of effective communication with researchers

### 2.4 Exclusion criteria

1. Refusal to provide informed consent.
2. Patients with neurological or psychiatric disorders that impede experimental cooperation, such as refractory epilepsy, Parkinson’s disease, and treatment-resistant depression
3. Abnormal ear anatomy
4. Severe cardiovascular and cerebrovascular disease (myocardial infarction within 12 months, NYHA heart rating ≥2, PaO2 < 60 mmHg)
5. History of pacemaker implantation.
6. Instrument allergy (including ear dermatitis)
7. Severe hepatic impairment (e.g., fulminant hepatitis resulting in Child-Pugh Class C) and renal dysfunction (e.g., serum creatinine >442 μmol/L, glomerular filtration rate <45 mL/min/1.73 m^²^). We supplemented the definitions of severe liver and kidney function disorders to further enhance the comprehensiveness of our research.
8. Previous partial hepatectomy
9. Biliary tract disease (calculi, infection, obstruction)

### 2.5 Dropout criteria

1. Voluntary withdrawal
2. Protocol violation
3. Concomitant medication interfering with trial outcomes
4. Loss to follow-up
5. Postoperative intensive care unit admission

Participants have the right to withdraw from the study at any time and for any reason without experiencing medical discrimination or disruption to their standard clinical care. The research team will ensure that all participants continue to receive appropriate medical care and will thoroughly document the reasons for withdrawal. Moreover, researchers may discontinue a participant’s involvement in the study if severe organ dysfunction, non-compliance with study protocols, or the occurrence of serious adverse events (SAEs) is observed.

### 2.6 Randomization and blinding

Participants will randomly be allocated to active-taVNS treatment or sham taVNS groups at a 1:1 ratio, based on a computer-generated random number sequence. The allocation process is strictly confidential and managed by administrative personnel uninvolved in study design and implementation. Each participant will be assigned a unique, de-identified alphanumeric code, encompassing the research center code, enrollment order, and randomization group identifier, ensuring data traceability. A designated research coordinator will maintain sealed, sequentially numbered opaque envelopes containing randomization assignments. Immediately before entering the operating room, a blinded circulating nurse will open the envelope to determine group assignment. Following verification of the group allocation details, the envelopes will be resealed, and this procedure will be conducted without disclosing the information to the patients. To ensure blinding, we will informed patients during preoperative consultation about potential sensory experiences during treatment, including sensations of pricking, needle-like stimulation, warmth, and vibration. We will explicitly emphasize that individual perception of stimuli varies significantly and that sensation intensity should not be used to assess treatment efficacy. Moreover, all stimulation devices will be designed with uniform appearance, with differences limited to electrical output parameters, thereby further preserving the integrity of the blinding procedure. The device will be preset to patient-specific maximum tolerable parameters by independent researchers not involved in the research, and will remain in a black screen state upon entering the operating room. All individuals involved in outcome assessment, data analysis, surgical procedures, and study participants remained blinded to group allocation. With regard to the initial administration of stimulation during hepatic portal occlusion, standard practice at this institution involves the surgeon informing the anesthesiologist and circulating nurse about surgical progress at this stage. However, these personnel were not provided with details regarding the specific stimulation protocol, thereby ensuring the maintenance of blinding within the surgical team throughout the procedure. Prior to study commencement, outcome assessors will accept rigorous blinding training, familiarizing themselves with assessment procedures and standards to minimize subjective influences on results.

### 2.7 Unblinding

During the intervention phase, participants will undergo systematic evaluation to identify potential complications associated with taVNS and life-threatening symptoms. Potential adverse events may include ear dermatitis, local bleeding at the stimulation site, ear pain, bradycardia, and cardiac arrhythmias. In the event of such complications, the unblinding procedure will be promptly initiated to minimize potential risks related to the intervention.

### 2.8 Anesthesia

Patients will undergo routine preoperative education and anesthesia consultation. During this period, we will collect relevant medical history, inform patients about anesthesia details and potential risks, and obtain informed consent for anesthesia and experimental procedures. Upon entering the operating room, peripheral venous access will be established, and monitoring of blood pressure, electrocardiogram, and pulse oxygen saturation will commence. Radial arterial and internal jugular venous catheters will be placed to monitor invasive arterial blood pressure and central venous pressure (maintained at <5 cm H_₂_O during surgery). Anesthesia induction will be achieved through intravenous administration of midazolam (0.05 mg/kg), etomidate (0.3 mg/kg), sufentanil (0.5 μg/kg), and cisatracurium (0.05 mg/kg), followed by rapid sequence intubation. After intubation, mechanical ventilation will be initiated with the following parameters: tidal volume 6–8 mL/kg, respiratory rate 12 breaths/min, inspiration-to-expiration ratio 1:1.5, inspired oxygen concentration 100%, oxygen flow rate 2 L/min, with end-tidal carbon dioxide partial pressure maintained between 35 and 45 mmHg. Anesthesia will be maintained using a combined intravenous and inhalational technique: continuous intravenous infusion of propofol at 4–6 mg/kg/h, remifentanil at 0.1–0.3 μg/kg/min, and 1% sevoflurane to maintain the bispectral index (BIS) between 40 and 60. Cisatracurium 0.05 mg/kg will be administered intermittently as needed. Vasoactive agents may be used to maintain heart rate and blood pressure within normal ranges. Tidal volume and respiratory rate will be adjusted to maintain end-tidal CO_₂_ between 35–45 mmHg. Fluid management will be tailored to intraoperative conditions, with central venous pressure maintained at <5 cm H_₂_O during hepatectomy. Sevoflurane will be discontinued 30 min before surgery’s end, and all anesthetic agents will be immediately stopped upon completion. The patient will be transferred to the post-anesthesia care unit (PACU). Postoperative patient-controlled analgesia (PCA) will contain sufentanil 1.5 μg/kg, tropisetron 6 mg, and normal saline 100 mL, with a continuous infusion rate of 2 mL/h, PCA dose of 0.5 mL, and a lockout interval of 15 min. Upon PACU arrival, atropine 0.5 mg and flumazenil 0.5 mg will be administered. Extubation will occur when the patient responds to verbal stimuli, muscle tone normalizes, and respiratory parameters are optimal. In the PACU, patients with a Numeric Rating Scale (NRS) pain score exceeding 5 may receive rescue analgesia. Patients with a Steward score greater than 4 can be transferred to the ward.

### 2.9 Study interventions

**Figure 2:**
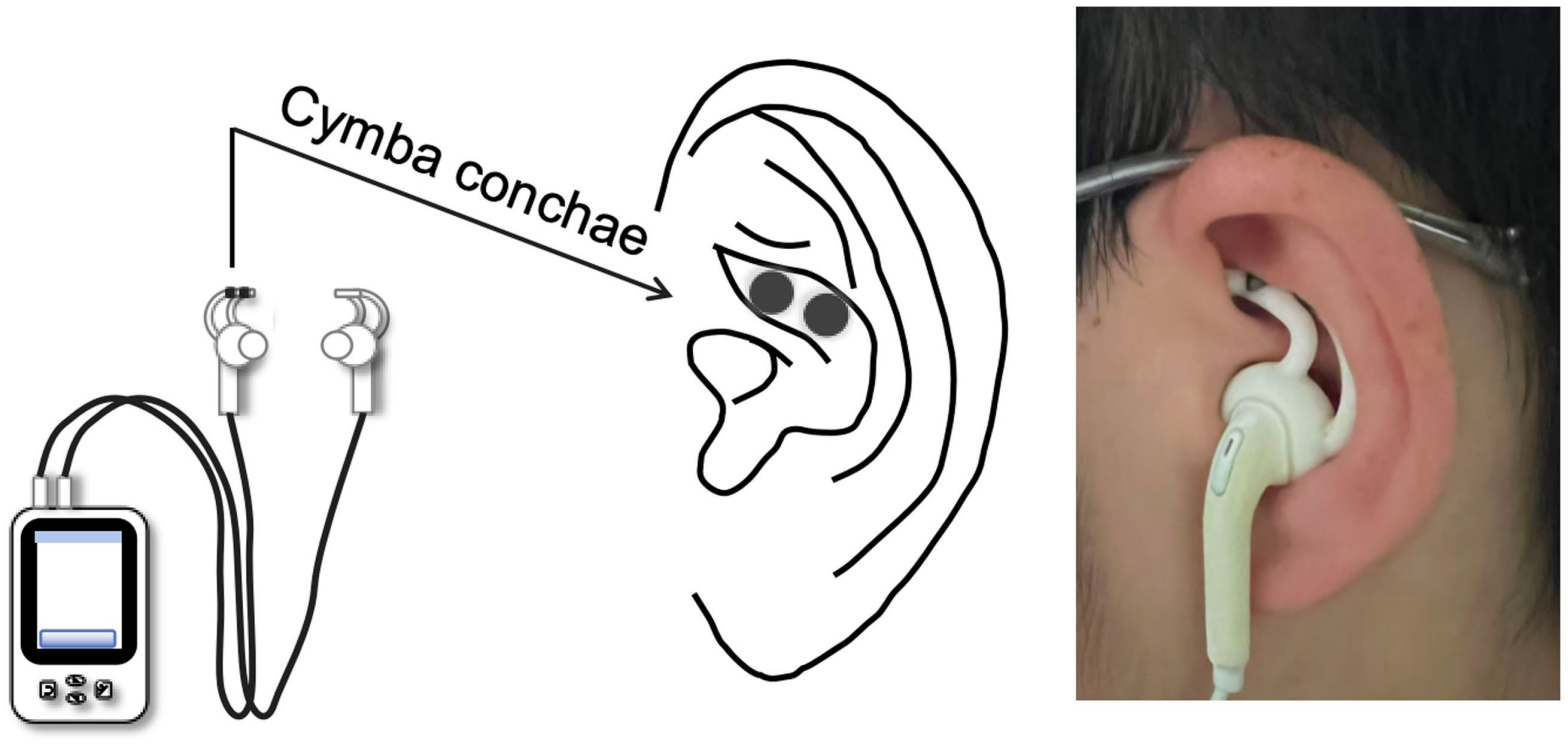
The stimulation electrode will be placed in the left ear position. taVNS, Transcutaneous auricular vagus nerve stimulation.

On the day before surgery, potential participants will be identified from elective surgical schedules. Active-taVNS or sham taVNS groups will be administered at four specific time points (as shown in Table 1): (1) at the onset of the first hepatic portal occlusion, (2) post-extubation, (3) on postoperative day 1 (6:00–7:00 a.m.), and (4) on postoperative day 2 (6:00–7:00 a.m.). taVNS will be performed using a transcutaneous electrical stimulation device (tVNS501, Reshen, Changzhou, China). Electrical stimulation will be applied via electrodes positioned near the auricular branch of the vagus nerve within the left ear cavity (as shown in Figure 2), a location demonstrated to optimally activate brain regions associated with organ control (30). Given the absence of clinical studies on taVNS’s hepatoprotective effects, stimulation parameters will be based on preclinical research (23). For active stimulation, before entering the operating room, independent researchers not involved in the research will calibrate the equipment, initiating stimulation amplitude at 0 (pulse width: 200 ms; frequency: 20 Hz) and incrementally increasing by 1 based on patient response. When participants report discomfort, display pain-indicative facial expressions, or when stimulation causes oxygen saturation to drop below 95% or heart rate to increase by more than 20%, the amplitude will be reduced to just below the previous threshold and maintained at a constant stimulus intensity output for 60 min. For the sham stimulation group, device placement and parameters remained consistent, with the sole difference being electrical current output (all device screens were adjusted and darkened before entering the room, and no current was applied in the sham group). The collection of the intensity of the first intervention will be conducted on the day before the surgery after the informed consent form is signed by the patient upon enrollment. Throughout the intervention, trained researchers strictly will adhere to the protocol and closely monitored participants’ responses to ensure no adverse reactions. If participants report intolerable discomfort, stimulation will be immediately terminated.

**Table 1:** The study schedule for enrollment, treatments, outcome measurements, and data collection.

Study period									
Timepoint	Enrollment	Allocation	Post-allocation						
Pre-2	0	Pre-1 (t0)	Pod-0 (t1-t2)	Pod-1 (t3)	Pod-2 (t4)	Pod-3 (t5)	Pod-5 (t6)	Pod-30 (t7)
Enrollment									
Inclusion criteria	☑								
Exclusion criteria	☑								
Informed consent	☑								
Randomization									
Allocation			☑						
Interventions									
Active-taVNS group				☑	☑	☑			
Sham taVNS group				☑	☑	☑			
Outcome measurement									
ALT			☑			☑		☑	
AST			☑			☑		☑	
TBA			☑			☑		☑	
DBIL			☑			☑		☑	
TBIL			☑			☑		☑	
BUN			☑			☑		☑	
Cr			☑			☑		☑	
IL-6			☑			☑			
TNF-α			☑			☑			
FRAIL			☑						
NRS					☑	☑		☑	☑
CAM			☑		☑	☑		☑	☑
SAS			☑		☑	☑		☑	☑
I-FEED					☑	☑	☑	☑	
FSS			☑		☑	☑		☑	☑
QoR-15			☑		☑	☑		☑	☑
Adverse events			☑	☑	☑	☑	☑	☑	☑

According to SPIRIT 2013 statement: defining standard protocol items for clinical trials. Pre, preoperative day; Pod, postoperative day; ALT, Alanine aminotransferase; AST, Aspartate aminotransferase; TBA, Total bile acids; TBIL, Total bilirubin; DBIL, Indirect bilirubin; BUN, Blood Urea nitrogen; Cr, Creatinine; IL-6, Interleukin-6; TNF-α, tumor necrosis factor-α; FRAIL, Fatigue, Resistance, Ambulation, Illness and Loss of Weight Index; NRS, Numerical Rating Scale; CAM, The Confusion Assessment Method; SAS, Simpson-Angus Scale; IEED, Intake, feeling nauseated, emesis, exam and duration of symptoms; FSS, Fatigue Severity Scale; QoR-15, Quality of Recovery-15.

### 2.10 Outcomes

#### 2.10.1 Primary outcome

The primary endpoint is serum alanine aminotransferase (ALT) levels on postoperative day 2. Perioperative hepatic injury occurs in two distinct phases. The first phase, occurring 2–6 h after reperfusion, is characterized by Kupffer cell-induced oxidative stress and the initiation of cell necrosis and apoptosis. The second phase, spanning 6–48 h after reperfusion, involves inflammatory cell aggregation in the hepatic microvascular system, including sinusoids and post-sinusoidal regions, accompanied by sinusoidal congestion, neutrophil adhesion leading to blood flow obstruction, and inflammatory cell extravasation into the hepatic parenchyma (31, 32). Furthermore, evidence indicates that intermittent stimulation effectively alleviates patient discomfort and improves treatment safety (33). Based on these pathological mechanisms, we implemented taVNS intervention throughout the entire hepatic injury inflammatory response to maximize inflammation mitigation and minimize hepatic damage. Liver injury severity is classified as follows: ① Mild acute liver injury (ALI) is typically defined as 2 × upper limit of normal (ULN) ≤ ALT < 5 ULN; ② Moderate ALI is typically defined as 5 ULN ≤ ALT < 15 ULN; ③ Severe ALI requires: International Normalized Ratio (INR) ≥ 2.0, ALT ≥ 10 ULN, serum total bilirubin (TBiL) ≥ 3.0 mg/dL, and absence of hepatic encephalopathy (34–36). Baseline venous blood samples will be obtained during the preoperative anesthesia visit after obtaining informed consent (t0). The primary endpoint will be venous blood samples collected at 7:00 AM on the second postoperative day (t4) after stimulation cessation.

#### 2.10.2 Secondary outcomes

1. ALT levels, will be measured on the fifth postoperative day (t6).
2. AST, total bile acids (TBA), total bilirubin (TBIL), direct bilirubin (DBIL), creatinine (Cr), and blood urea nitrogen (BUN) levels, will be measured at 7:00 a.m. on the second and fifth postoperative days (t4 and t6).
3. Interleukin-6 (IL-6) and TNF-*α* levels, will be measured at 7:00 a.m. on the second postoperative day (t4).
4. Postoperative recovery quality, will be assessed using the Postoperative Quality of Recovery-15 (QoR-15) scale on the first, second, fifth, and thirtieth postoperative days (t3-t4 and t6-t7). The QoR-15 scale evaluates 15 items across physical adaptability, self-care ability, psychological state, and postoperative pain, with scores ranging from 0 to 10 (total score 150); lower scores indicate poorer postoperative recovery (37).
5. Anxiety levels will be evaluated using the Self-Rating Anxiety Scale (SAS) on the first, second, fifth, and thirtieth postoperative days (t3-t4 and t6-t7). The SAS comprises 20 items scored 1–4, with a raw score range of 20–80. The standard score is calculated by multiplying the raw score by 1.25, yielding a total score of 25–100. Anxiety is defined as a SAS score ≥50, with severity categorized as: 25–49 (no anxiety), 50–59 (mild anxiety), 60–69 (moderate anxiety), and 70–100 (severe anxiety) (38).
6. Pain intensity will be measured using the NRS on the first, second, fifth, and thirtieth postoperative days (t3-t4 and t6-t7), ranging from 0 (no pain) to 10 (most severe pain imaginable) (39).
7. The incidence of postoperative delirium will be evaluated using the Confusion assessment method (CAM) scale on the first, second, fifth, and thirtieth postoperative days (t3-t4 and t6-t7) (40).
8. Fatigue levels will be assessed using the Fatigue Severity Scale (FSS) on the first, second, fifth, and thirtieth postoperative days (t3-t4 and t6-t7). The FSS comprises nine statements scored 1–7, with a total score range of 9–63. Higher scores indicate more severe fatigue, with ≥ 36 signifying significant fatigue symptoms (41).
9. Postoperative gastrointestinal function recovery will be evaluated using the Intake-Food Intolerance-Emesis-Dyspepsia-Endoscopy-Duration (I-FEED) scoring system on the first, second, third and fifth postoperative days (t3-t6), Some patients with severe gastrointestinal dysfunction (Postoperative Gastrointestinal Dysfunction, POGD) were followed up until the end of the surgery. I-FEED, recommended by the American Society for Enhanced Recovery and Perioperative Quality, is the primary method for diagnosing POGD. The scoring system classifies gastrointestinal function as follows: 0–2 points indicate normal function; 3–5 points suggest intolerance; ≥ 6 points signify POGD (42).
10. Time to first flatus, defecation, and oral intake will be recorded.

### 2.11 Adverse events and safety

Potential adverse events (AEs) related to electrical stimulation, including skin allergy, tingling, dizziness, nausea, and vomiting, will be closely monitored. AEs and SAEs will be meticulously documented, appropriately managed, and tracked until resolution or stabilization, with a comprehensive assessment of their causal relationships. Data on all AEs and SAEs will be separately coded, listed, and reported in future publications. SAEs and unexpected events will be promptly reported to the ethics committee and regulatory authorities as required, who retain the ultimate decision to terminate the trial. The risk of bias in this study is low; in the event of AEs, independent data monitoring will be arranged to collect research data. Concurrently, given the potential risk of experimental invalidity, the Data Monitoring Committee will periodically review cumulative safety and efficacy data, conducting an interim analysis when approximately 50% of data collection is complete. This analysis will assess whether statistically significant differences exist between the two groups (*p* < 0.05), comprehensively evaluating study progress and providing recommendations regarding study continuation. During intervention, if an adverse reaction is suspected, the research device can be Data will be collected at three time points: preoperative, intraoperative, and postoperative immediately discontinued, and unblinding will be swiftly implemented to ensure participant safety.

### 2.12 Data collection and management

These data will be recorded in a case report form (CRF). Preoperative information includes comprehensive demographic data, medical history, current health status, surgical type, nutritional control status scoring (including preoperative hemoglobin levels and lymphocyte count), CAM, SAS, FRAIL and FSS scores. Additionally, preoperative liver and kidney function indicators, coagulation function parameters and inflammatory markers will be collected. Intraoperative data will encompass detailed metrics such as blood loss, urine output, ischemic occlusion frequency and duration, mean arterial pressure (MAP) and central venous pressure (CVP) during occlusion and reperfusion phases, extent of hepatectomy [Hepatectomy is classified as minor when involving up to four wedge resections or two segmental resections, and major when exceeding these criteria, such as left or right hemihepatectomy (43)], detailed medication records, and surgical and anesthesia duration. Postoperative data will include liver and kidney function, inflammatory markers, NRS for pain intensity, QoR-15 for overall recovery assessment, CAM and FSS scores for delirium and fatigue incidence, and gastrointestinal function recovery data.

After the trial, all participant data will be analyzed by an independent, professionally trained investigator who was not involved in any aspect of the experiment. Personnel responsible for perioperative follow-up and blood sample collection were blinded to group allocation and will not participate in interventions. Following sample collection, specimens will be centrifuged, classified, and recorded in an encrypted Excel spreadsheet stored on an iPad. Test results will be subsequently entered into the same Excel file. An independent investigator collected pre-, intra-, and post-operative questionnaire data, initially recording them on paper case report forms, then transcribing them into a Word document on the iPad. In accordance with national regulatory requirements, original samples will be stored in a dedicated anesthesia freezer pending authorized access. The iPad containing follow-up information, case report forms, and other sensitive data will be password-encrypted and secured in a locked cabinet, accessible only to authorized personnel for data entry, processing, and analysis.

### 2.13 Sample size

Sample size was calculated using PASS 15.0 software. The primary outcome measure was ALT level on postoperative day 2. Preliminary data showed a mean ALT level of 249.8 ± 147.9 U/L after partial hepatectomy. In a pre-experimental phase, taVNS interventions reduced ALT to 174.9 ± 103.5 U/L (approximately 30% reduction). With a significance level (*α*) of 0.05 and statistical power (1 − *β*) of 90%, a two-sided test determined a minimum sample size of 63 participants per group. Accounting for a 10% potential dropout rate, we plan to recruit 140 patients and randomly allocate them to active-taVNS and sham taVNS groups. Regarding the validation of the Minimal Clinically Important Difference (MCID), we estimated the MCID using a stepwise approach based on previous research methods (44). In this experiment, the sham taVNS group’s mean ALT ± standard deviation on postoperative day 2 was 249.8 ± 147.9 U/L, while the active-taVNS group was 174.9 ± 103.5 U/L, with a pooled standard deviation of 127.64. Conventionally, the MCID range is typically set between 0.2 and 0.5 times the pooled standard deviation, resulting in a reasonable MCID interval of 25.53 to 63.82. The mean difference between groups (*Δ*) was 74.9. When the intergroup difference exceeds the upper limit of the MCID, it indicates that the observed effect difference has clinical significance, thus supporting the reasonableness of the MCID determination.

### 2.14 Statistical analysis

Statistical analyses will be performed using SPSS software (version 27.0; IBM) and statistical charts will be generated with GraphPad Prism (version 8.0). Normality of data will be assessed using the Kolmogorov–Smirnov test, and variance homogeneity will be evaluated by the Levene test. Normally distributed continuous data will be presented as mean ± standard deviation, while non-normally distributed data will be reported as median with interquartile range.

Statistical analyses will employ independent samples t-tests for normally distributed continuous data and Mann–Whitney U tests for non-normally distributed data. Time-to-event outcomes will be evaluated using Kaplan–Meier curves and Log-rank tests. Multivariate analyses will utilize multiple linear or logistic regression models based on dependent variable type. Repeated measures data will be analyzed using generalized estimating equations. The analysis will follow the intention-to-treat principle, with multiple imputation addressing missing data the per-protocol Analysis will be included as part of the sensitivity analysis. A significance level of *α* = 0.05 will be applied, with *p* < 0.05 considered statistically significant.

## 3 Discussion

Partial hepatectomy remains the primary surgical treatment for primary and metastatic liver tumors (5). However, several perioperative risk factors can negatively affect liver function. From an anatomical and physiological perspective, the separation of liver parenchyma results in substantial microscopic and organ-level alterations in perfusion, directly impacting the liver’s blood supply and drainage system (8). Massive intraoperative blood loss may trigger fluid redistribution and systemic inflammatory responses, thereby substantially increasing the risk of postoperative infections (45, 46). Moreover, evidence suggests that extensive hepatectomy and the presence of hepatocellular carcinoma are key risk factors for postoperative coagulopathy and the development of a hypercoagulable state (47). Although the Pringle maneuver is commonly employed for intermittent occlusion of the portal vein to control intraoperative bleeding, prolonged occlusion may exacerbate HIRI and further impair liver function (48). Studies have shown that HIRI can lead to liver failure and systemic inflammatory response syndrome (SIRS), which may reduce postoperative tumor-free survival rates and elevate the risk of tumor recurrence (9, 49, 50). Furthermore, preclinical studies have demonstrated that chronic stress negatively affects liver regeneration and is associated with increases mortality following partial hepatectomy in mice (51). These findings are corroborated by population-based studies, which show that elevated levels of psychological stress are linked to higher mortality rates among patients with liver disease (52). Perioperative liver protection has therefore emerged as a key area of focus in the fields of in anesthesiology and surgical practice. Traditional hepatoprotective strategies during the perioperative period include ischemic preconditioning and anesthetic preconditioning. Although clinical evidence suggests that ischemic preconditioning can significantly reduce postoperative transaminase levels, its clinical applicability is limited by the complexity of the procedure, which involves repeated clamping of the portal vein and may potentially cause damage to small portal vein branches (53). A recent meta-analysis challenges the efficacy of ischemic preconditioning in reducing liver damage caused by ischemia–reperfusion injury during hepatectomy, thereby challenging its hepatoprotective benefits in the perioperative setting (12). The hepatoprotective effects of propofol remain a subject of debate. Although certain studies suggest that propofol anesthesia may significantly reduce postoperative ALT levels and decrease the incidence of complications, a meta-analysis has also identified a potential link between propofol and drug-induced liver injury (54, 55). Opioids have shown promise in ameliorating hepatic ischemia–reperfusion injury through their anti-inflammatory properties and modulation of vagal nerve activity. However, their use is accompanied by a risk of respiratory depression, which warrants cautious application in clinical practice (56). taVNS, as a novel perioperative strategy for liver protection, is characterized by its notable for its ease of application, minimal side effects, and well-established theoretical basis. VNS has been confirmed to attenuate perioperative hepatic injury through several mechanisms, including upregulation of *α*-7nAChR expression, activation of the CAP, and elevation of glutathione levels (19–21). Recent studies have indicated that vagal hepatic branches play a crucial role in promoting liver regeneration. Liu et al. demonstrated that activation of vagus nerve enhances IL-6 signaling in hepatic macrophages, thereby promoting hepatocyte proliferation and regeneration (57). Moreover, VNS has exhibited promising potential in suppressing tumor growth and metastasis. In a mouse model of colorectal cancer with liver metastasis, selective deactivation of the vagus nerve was associated with a significant increase in the number of hepatic metastatic nodules, higher mortality rates, and larger tumor volumes compared to those observed in the sham-operated group (58). Finally, research indicates that VNS can induce the release of acetylcholine (Ach) through the spleen, which subsequently interacts with the *α*-7nAChR on T lymphocytes and platelets, thereby promoting calcium ion influx, enhancing α-granule release, and activating circulating platelets to increase local thrombin generation. As a result, this mechanism accelerates the coagulation cascade and reduces hemorrhage at the site of tissue injury, thereby improving patient recovery outcomes (59). These findings suggest that VNS may function as a comprehensive, multidimensional perioperative strategy for hepatoprotection, capable of attenuating hepatic injury, decreasing perioperative blood loss, suppressing tumor progression, and facilitating liver regeneration. In terms of safety, a systematic review of available literature on taVNS, not restricted to cardiovascular applications, revealed that the most frequently reported adverse events were localized stimulation-related symptoms such as pricking sensations, pain, swelling, or itching, occurring in 16.7% of cases. Additional adverse events included headache (3.3%), nasopharyngitis (1.6%), and dizziness or syncope (1.4%). Importantly, only three serious adverse events were considered directly attributable to taVNS (60). VNS primarily comprises two modalities: implanted VNS (iVNS) and transcutaneous VNS (tVNS). The latter includes both taVNS and transcutaneous cervical VNS (61). iVNS is associated with potential complications such as delayed bradycardia, severe cardiac arrest, including bradycardia, peritracheal hematoma, and atrioventricular block. Furthermore, its clinical application is significantly constrained by high implantation costs (62–64). Due to its favorable safety profile, low cost, and ease of access, taVNS has increasingly been considered a promising first-line therapeutic option (65). Previous clinical studies have demonstrated that non-invasive VNS exhibits efficacy comparable to that of traditional implantable VNS (66).

Currently, both clinical practice and scientific research predominantly focus on individual diseases or physiological systems. However, the prevailing disease-oriented diagnostic and therapeutic paradigm requires urgent reevaluation (67). Complex comorbidities involving central and peripheral systems are common in clinical settings, often interacting in ways that exacerbate and accelerate disease progression (67). A growing body of evidence indicates that perioperative factors, such as anxiety, preoperative fasting, and postoperative pain, can negatively influence surgical outcomes and contribute to increased mortality among patients with liver disease (52, 68, 69). Moreover, the prevalence of depression is significantly elevated in individuals with chronic liver disease. Hepatic injury induced by ischemia–reperfusion during partial hepatectomy can lead to systemic inflammation, which may in turn trigger depressive-like phenotypes (70). In a 2024 review titled “Transcutaneous auricular Vagus Nerve Stimulation as a Novel Therapy Connecting Central and Peripheral Systems,” it was highlighted that taVNS therapy is widely utilized in the regulation of central nervous system disorders, including epilepsy, depression, insomnia, migraine, anxiety, phobia, cognitive impairment, post-traumatic stress disorder, Parkinson’s disease, disorders of consciousness, and cognitive decline (71, 72). Furthermore, taVNS has been shown to modulate the function of peripheral organs, including the heart, lungs, liver, pancreas, gastrointestinal tract, and spleen (67). Consequently, taVNS therapy presents a promising therapeutic approach for conditions arising from autonomic dysfunction in both central and peripheral nervous systems, providing a novel strategy for addressing comorbidities (67).

ALT is a well-recognized biomarker for liver injury. Compared to AST, ALT exhibits higher specificity in identifying hepatocellular damage and is commonly included in standard metabolic panels as a key indicator of hepatic dysfunction. ALT activity primarily found in the cytoplasm of hepatocytes, where intracellular concentrations are approximately 3,000 times greater than those in the serum. As a result, both acute and chronic hepatocyte injury can significantly increase serum ALT levels due to the release of this enzyme from damaged or necrotic cells (73, 74). ALT has been widely utilized as a primary endpoint in numerous preclinical and clinical studies assessing interventions for liver injury. With a half-life of approximately 48 h and its relevance to the intervention period extending through postoperative day two, ALT serves as a suitable and reliable primary outcome measure for the present study (73, 75, 76).

In the absence of prior research on the hepatoprotective effects of taVNS, we determined our stimulation parameters based on preclinical evidence derived from traditional VNS studies (23). A systematic review of 33,531 taVNS-related studies indicated that 74% utilized a stimulation frequency of 20 Hz, with more than half employing a pulse width of 200 μs. Taking both safety and efficacy into account, we selected a stimulation protocol of 20 Hz and 200 μs (77).

Several limitations of this study should be acknowledged. First, as a single-center study with a relatively modest sample size, the generalizability of the findings may be limited. Second, the restricted variety of hepatectomy types and the limited number of liver transplant procedures conducted at our institution may constrain the broader applicability of the results. Concerning the intervention protocol, we applied fixed stimulation parameters without systematically investigating potential dose–response relationships across different parameter combinations. Finally, although we will assess levels of inflammatory cytokine such as IL-6 and TNF-*α*, we will not directly measure upstream indicators such as vagal nerve activity or α-7nAChR expression, thereby leaving the activation mechanism of the cholinergic anti-inflammatory pathway at a speculative level.

Although preclinical studies on taVNS for hepatic protection during the perioperative period are emerging, clinical evidence remains limited. This study examines the effect of this non-invasive neuromodulation technique on postoperative liver function in patients undergoing elective partial hepatectomy. By introducing this novel therapeutic approach, the research provides a new perspective on multimodal perioperative hepatoprotection and may contribute to the advancement of organ preservation strategies.

## Glossary

taVNS: Transcutaneous auricular vagus nerve stimulation
HIRI: Liver ischemia–reperfusion injury
VNS: Vagus nerve stimulation
iVNS: Vasive vagus nerve stimulation
*α*-7nAchR: Alpha-7-nikotinische Acetylcholinrezeptoren
CAP: Cholinergic anti-inflammatory pathway
TNF-α: Tumor necrosis factor-α
ALT: Alanine aminotransferase
AST: Aspartate aminotransferase
TBA: Total Bile Acid
DBIL: Direct Bilirubin
TBIL: Total Bilirubin
BUN: Blood Urea Nitrogen
Cr: Creatinine
IL-6: Interleukin-6
APTT: Partial thromboplastin time
PT: Prothrombin time
INR: International normalized ratio
SIRS: Systemic inflammatory response syndrome
BIS: Bispectral index
PACU: Post-anesthesia care unit
PCA: Patient-controlled analgesia
SAS: Simpson-Angus Scale
CAM: Confusion Assessment Method
FSS: Fatigue Severity Scale
NRS: Numerical Rating Scale
QoR-15: Quality of Recovery-15
FRAIL: Fatigue, Resistance, Ambulation, Illness and Loss of Weight Index
IEED: Intake, feeling nauseated, emesis, exam and duration of symptoms scoring system
SAEs: Serious adverse events
AEs: Adverse events
