# Supplementary material for: Effect of transcutaneous auricular vagus nerve stimulation on postoperative liver function in patients undergoing partial hepatectomy: a study protocol for a prospective, double-blind, randomized controlled trial
Source: Front Med (Lausanne). 2025 Aug 12;12:1603543. doi: 10.3389/fmed.2025.1603543 (PMC12378827; doi:10.3389/fmed.2025.1603543)
Supplement: Supplementary file 2 [file Table_2.DOCX]

Informed consent

Dear Patient:

We sincerely invite you to participate in a clinical study titled "The Impact of Transcutaneous Vagus Nerve Stimulation on Postoperative Liver Function Protection in Patients Undergoing Partial Hepatectomy". Before you decide whether to participate in this study, please read the following carefully to help you understand the study's purpose, process and duration, as well as the potential benefits, risks and inconveniences that may result from your participation. If you have any questions or do not understand something, please consult the researcher (doctor) until you are fully clear. You can also discuss with your family, relatives, friends, etc. to help you make the decision that best suits your interests.

The following is an introduction to the study: I. Background and purpose of the research

Postoperative liver function impairment is one of the most common and serious complications after partial hepatectomy. However, due to the complexity of surgical procedures and intraoperative ischemia-reperfusion injury, patients with poor prognosis of postoperative liver function have increased perioperative complications, which prompts us to explore new therapies that can effectively protect postoperative liver function and improve the quality of life of patients after surgery. Therefore, this study attempts to apply taVNS technology to patients undergoing hepatobiliary surgery to evaluate the effect of taVNS on postoperative liver function protection in patients undergoing partial hepatectomy, and to observe whether taVNS intervention can effectively protect postoperative liver function by alleviating perioperative inflammation and liver injury caused by various mechanisms.

II. Specific procedures and procedures

According to the inclusion and exclusion criteria, eligible and willing study participants were screened, provided their study details, voluntarily selected and signed informed consent forms, checked basic information, recorded relevant medical history, and wore the taVNS device at four specific time points: (1) at the onset of the first hepatic portal occlusion, (2) after extubation, and (3) from 6:00 to 7:00 am on the first postoperative day: (4) The device was connected according to the enrollment situation at 6:00 to 7:00 am on the second postoperative day, and the pulse frequency was set to 20Hz. The pulse width was 200us (pulse amplitude was defined as the presence of current but no obvious stabbing pain), and electrical stimulation was performed for 60 min. After the end of treatment, four planned follow-up visits were scheduled over a period of 1 month.

III 、What do you need to do if you participate in the study

1. You need to understand the specific situation of this study in detail, under the

premise of full understanding, voluntarily choose whether to participate in this study. If you agree to participate in this study, you need to sign this informed consent, and

agree to our use of your medical information.

2. Cooperate with us for related operations and postoperative follow-up. IV. Benefits that may be brought to you by participating in this study

By participating in this study, you may reduce postoperative pain, nausea and vomiting, and improve the quality of postoperative recovery from postoperative liver injury. You may not benefit from this study.

However, according to your data during the study period, researchers can further understand the role of auricular vagus nerve in the protection of postoperative liver function, and choose a more reasonable perioperative period to reduce perioperative liver injury, which will bring benefits to your future diagnosis and treatment and the same patients. There was no compensation for participation in the program.

V. Possible adverse reactions, risks, prevention and treatment measures of participating in this study

If dermatitis occurs during the intervention period, the stimulation should be stopped, observation should be carried out, and no improvement should be made. Dermatology consultation should be conducted and drug treatment should be given; If there is local bleeding at the stimulation site, stop stimulation, press to stop bleeding, and disinfect; If ear pain occurs, stop stimulation, observation, no improvement, please consult the ear, nose and throat department for treatment; During the use of taVNS instrument, ECG monitoring will be carried out to observe the skin around the irritation; If there is any damage related to the study, it will be actively treated, and relevant experts will be consulted. The expenses incurred will be borne by the Department of Anesthesiology.

VI. Description of expenses

1. Preoperative examination, anesthesia, operation and postoperative treatment are all necessary for your normal diagnosis and treatment, and the costs incurred shall be borne by you;

2. The expenses for the purchase and use of taVNS instruments are borne by our research group.

VII. Alternative plan

This study is an interventional study. Without participating in this study, we will conduct it according to perioperative anesthesia and surgical procedures. If adverse reactions occur after surgery, they will be dealt with by clinicians according to clinical guidelines.

VIII 、Your Rights

Participation in the study is entirely voluntary. You may decline to participate in the study, or withdraw from the study without reason at any time during the study, without affecting your relationship with your doctor, or the loss of medical or other benefits to you. If you have any questions about this study or during the study, please contact Yuanyuan Wang, Investigator of this study at 17354228367.

IX. Confidentiality of your personal information

Your medical records (including research records and physical and chemical

examination reports, etc.) will be kept in the hospital as required. Except for relevant personnel such as researchers, ethics committees, supervisors, inspectors, and drug administration departments, other personnel not related to the study have no right to access your medical records without permission. Your personal information will not be disclosed in the public report of the results of this study. We will make every effort to protect the privacy of your personal medical information to the extent permitted.

X. Termination of study participation

Your participation in the study may be terminated for the following reasons:

1. You did not follow the study doctor's orders.

2. You have a serious condition that may require treatment.

3. The study doctor believes that terminating the study is in the best interest of your health.

4. You withdraw your consent XI. Ethics Committee

This study has been reported to the Ethics Committee of the Affiliated Hospital of Xuzhou Medical University, and was approved after review by the committee. During the study process, the Ethics Committee of the Affiliated Hospital of Xuzhou Medical University can be contacted for ethics and rights issues.

Tel: +86 516 8580 2291 Email Address: [hzszxyyll@163.com](mailto:hzsyll@163.com)


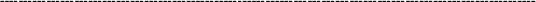


**Researcher statement:**

I confirm that I have explained to the subject the details of the study, including its rights and possible benefits and risks, answered the subject's questions, and the subject stated that he understood my explanations and explanations. I have provided a signed copy of the informed consent to the subject.

Investigator Signature: Investigator Contact Number:

Date of conversation: at

**Subject statement:**

I confirm that I have read the informed consent for this study, and the researcher has explained the relevant contents to me in detail, answered my questions, and made me understand the terms and conditions therein. I also confirm that if I do not participate in this study or withdraw at any time during the study, my diagnosis and treatment activities and doctor-patient relationship will not be affected, or other interests will be harmed. I understand that the public reporting of the results of this study will not disclose my personal information. I have had plenty of time to think about it. After careful consideration, I have decided to accept the treatment (research) approach in this study and agree to use my relevant research data and information for public reporting related to the results of this study.

Subject signature: Signature date:

Subject's contact phone number:
